# Hard–Soft Core–Shell Architecture Formation from Cubic Cobalt Ferrite Nanoparticles

**DOI:** 10.3390/nano13101679

**Published:** 2023-05-19

**Authors:** Marco Sanna Angotzi, Valentina Mameli, Dominika Zákutná, Fausto Secci, Huolin L. Xin, Carla Cannas

**Affiliations:** 1 Department of Chemical and Geological Sciences, University of Cagliari, Cittadella Universitaria S.S. 554 Bivio per Sestu, 09042 Monserrato, Italy; marcosanna@unica.it (M.S.A.); fausto.secci@unica.it (F.S.); 2 Consorzio Interuniversitario Nazionale per la Scienza e Tecnologia dei Materiali (INSTM), Via Giuseppe Giusti 9, 50121 Florence, Italy; 3 Department of Inorganic Chemistry, Charles University, Hlavova 2030, 128 40 Prague 2, Czech Republic; dominika.zakutna@natur.cuni.cz; 4 Department of Physics and Astronomy, University of California, Irvine, CA 92617, USA; huolinx@uci.edu

**Keywords:** cobalt ferrite, core–shell, heterostructures, cubic shape, STEM-EDX

## Abstract

Cubic bi-magnetic hard–soft core–shell nanoarchitectures were prepared starting from cobalt ferrite nanoparticles, prevalently with cubic shape, as seeds to grow a manganese ferrite shell. The combined use of direct (nanoscale chemical mapping via STEM-EDX) and indirect (DC magnetometry) tools was adopted to verify the formation of the heterostructures at the nanoscale and bulk level, respectively. The results showed the obtainment of core–shell NPs (CoFe_2_O_4_@MnFe_2_O_4_) with a thin shell (heterogenous nucleation). In addition, manganese ferrite was found to homogeneously nucleate to form a secondary nanoparticle population (homogenous nucleation). This study shed light on the competitive formation mechanism of homogenous and heterogenous nucleation, suggesting the existence of a critical size, beyond which, phase separation occurs and seeds are no longer available in the reaction medium for heterogenous nucleation. These findings may allow one to tailor the synthesis process in order to achieve better control of the materials’ features affecting the magnetic behaviour, and consequently, the performances as heat mediators or components for data storage devices.

## 1. Introduction

In the field of nanotechnology, the combination of different materials for the obtainment of heterostructured multifunctional nanoparticles (NPs) [1,2,3] or nanocomposites [4,5,6,7,8] has driven the efforts of researchers with the aim of exploiting the physical (magnetic, optical, electrical, etc.) and chemical (thermal stability, reactivity, bonding ability, dispersibility, solubility, etc.) properties of single components in a combined or synergistic manner for selected applications. When two (or more) different phases are put in contact at the nanoscale, new phenomena may arise from their coupling, with a crucial role played by the interface extent. One of the most prominent examples is core–shell nanoarchitectures, consisting of an inner core covered by an external layer (shell) of a different material. The nature of the contact between the core and the shell is fundamental for these structures to show their peculiar properties; particularly, a net change in the chemical composition with no chemically mixed layers between the phases is desired. Diverse materials such as ferrites, perovskites, silica, titania, noble metals, elemental carbon, alloys, and polymers have been coupled to form inorganic/organic or inorganic/inorganic core–shell systems [9]. The choice of the materials to be coupled, the core–shell volume ratio, and the synthesis method are elements to play with. Core–shell heterostructures featuring a heterojunction between two different materials have an extensive range of applications, such as photocatalysis [10,11], gas sensing [12,13], water purification [14], and hydrogen production [15,16]. In these materials, the electron phenomena taking place at the interface between the two phases play a crucial role [17,18].

In the case of magnetic materials, since the discovery of exchange bias phenomena in a ferromagnet (FM)/antiferromagnet (AFM) system (Co/CoO core–shell NPs) by Meiklejohn and Bean [19], and by Kneller and Hawig [20], other interfaces have been built, such as AFM/ferrimagnet (FiM), or hard–soft FM or FiM [21,22,23,24,25]. The development of the core–shell heterostructure is favoured by the choice of isostructural crystalline phases, which should grow epitaxially one upon the other. In this context, spinel ferrite (M^II^Fe_2_O_4_, M^II^ = Fe^2+^, Co^2+^, Mn^2+^, Ni^2+^, Zn^2+^, etc.) bi-magnetic core–shell NPs have been studied and proposed in different application fields, such as magnetic recording, permanent magnets, spintronics, microwave absorption, biomedicine, magnetic heat generation for catalysis [26,27,28,29], or magnetic fluid hyperthermia (MFH) [30,31,32,33,34,35,36,37,38,39,40,41,42,43,44,45]. In this framework, the adoption of core–shell heterostructures was demonstrated to be efficient to improve the heating abilities of spinel ferrites or their energy product as permanent magnets [21,25,32,39,46]. The spinel ferrite core–shell NPs offer a tuneable range of hard and soft magnetic behaviours by properly changing the chemical composition. They can be successfully prepared with good crystallinity and good control over the composition, the particle’s morphology, and heterostructure interface by seed-mediated approaches applied to thermal decomposition and solvothermal methods [32,39,47,48,49,50,51,52,53,54,55,56,57,58,59,60,61].

Seed-mediated synthesis approaches consist of introducing pre-formed nanoparticles (seeds) into the medium containing the precursors of the shell material. The nucleation of the secondary material onto the surface of the seeds (heterogeneous nucleation) has a lower energy barrier than the formation of new particles of the secondary material (homogeneous nucleation), thus giving rise to the formation of the core–shell structures [62].

In particular, the magnetic behaviour of the core–shell NPs can also be modified according to the shape, in addition to the size, for instance, due to surface anisotropy effects. Regarding this effect, a remarkable influence of the NPs’ shape on their magnetic properties was observed by several authors [9,63,64,65,66,67,68,69]. Concentric spherical core–shell NPs are the most common systems [9].

Recently, we set up a versatile solvothermal synthesis method able to produce single-phase spherical particles [70,71], chemically mixed spinel phases [72,73], homogeneous spherical bi-magnetic core–shell NPs [46,70,74,75], and flower-like Ag-spinel ferrite nanoarchitectures [1] with high crystallinity, low size dispersity, and precise control of the shell growth. 

Herein, we studied the formation of cubic cobalt ferrite nanoparticles and their bi-magnetic heterostructure counterparts by coating cubic cobalt ferrite nanoseeds with manganese ferrite to form CoFe_2_O_4_@MnFe_2_O_4_ with the same oleate-based solvothermal method adopted in our previous studies. High-resolution transmission electron microscopy (HRTEM) and chemical mapping at the nanoscale, performed through STEM-EDX, together with the Rietveld refinement of X-ray diffraction (XRD) patterns and DC magnetometry, were employed to study morphological, structural, and magnetic properties.

The study aimed to provide precious details on the formation mechanism of core–shell heterostructures by starting from larger (15 nm versus 6–8 nm) and anisotropic (cubic versus spherical) cobalt ferrite nanoparticles compared with our previous studies. To understand how the different morphologies of the seeds affect the final product, a combination of direct microscopic and indirect macroscopic characterisation techniques was adopted. The chemical mapping at the nanoscale via STEM-EDX allowed us to probe the local chemical composition of a few nanoparticles with a good resolution, while DC magnetometry provided a bulk-level view of the average physical behaviour, beyond the representativeness limits of electron microscopy.

## 2. Materials and Methods

### 2.1. Chemicals

Oleic acid (>99.99%), 1-pentanol (99.89%), hexane (84.67%), and toluene (99.26%) were purchased from Lach-Ner, Neratovice, Czech Republic; 1-octanol (>99.99%) and Mn(NO_3_)_2_·4H_2_O (>97.0%) from Sigma-Aldrich, St. Louis, MO, USA; absolute ethanol and Co(NO_3_)_2_·6H_2_O (99.0%) from Penta, Prague 10, Czech Republic; NaOH (>98.0%) from Fluka, Muskegon, MI, USA; Fe(NO_3_)_3_·9H_2_O (98.0%) from Lachema, Brno, Czech Republic.

### 2.2. Methods

Cobalt ferrite NPs and core–shells were prepared as described in previous work [46,70], starting from metal oleates. The synthesis conditions are summarised in Table S1.

### 2.3. Characterisation

Fourier Transform Infrared (FT-IR) spectra were recorded in the region from 400 to 4000 cm^−1^ by using a FT/IR-4X Spectrometer from Jasco, Easton, MD, USA. Samples were measured in a KBr pellet.

Thermogravimetric analysis (TGA) curves were obtained by using a PerkinElmer (Waltham, MA, USA) STA 6000, in the 25–850 °C range, with a heating rate of 10 °C min^−1^ under 40 mL min^−1^ O_2_ flow.

The samples were characterised via X-ray diffraction (XRD), using a PANalytical X’Pert PRO (Malvern PANalytical, Malvern, UK) with Cu Kα radiation (1.5418 Å), a secondary monochromator, and a PIXcel position-sensitive detector. The peak position and instrumental width were calibrated using powder LaB_6_ from NIST. The refinement of the structural parameters was carried out using the Rietveld method in the FullProf software [76] using a pseudo-Voigt profile function. For mean crystallite shapes, the spherical harmonics function was used [77]:

(1) $$T\left(h\right)=T\left(\vartheta ,\varphi \right)=\overset{n}{\underset{l=0,2,4\ldots }{\sum }}\overset{l}{\underset{m=-l}{\sum }}a_{l,m}Y_{l,m}\left(\vartheta ,\varphi \right)$$

where ϑ and φ are polar and azimuthal angles describing the direction of the normal to the family of the lattice plane in a Cartesian coordinate system, a is the lattice parameter, and Y is the Lorentzian isotropic size broadening.

TEM micrographs were acquired using a JEOL 200CX electron microscope (Jeol Ltd., Tokyo, Japan) operating at 160 kV. The size of more than 1000 particles was measured using Pebbles software [78], selecting a cubic or spherical shape in order to determine the particle size distribution. The average particle diameter was calculated together with the percentage ratio between the standard deviation and the mean value to provide the size dispersity. Additional TEM micrographs and EDX data for the Co:Mn:Fe molar ratios were obtained by using a JEOL JEM 1400 Plus (Jeol Ltd., Tokyo, Japan) operating at 120 kV.

HRTEM micrographs and STEM-EDX measurements were carried out using an FEI Talos F200X (Thermo Fisher Scientific, Waltham, MA, USA) equipped with a field-emission gun operating at 200 kV and a four-quadrant 0.9 sr energy-dispersive X-ray spectrometer.

The Quantum Design PPMS DynaCool system with a maximum magnetic field of 9 T and the VSM module was used to investigate the DC magnetic properties of powders. The magnetisation values were normalised based on thermogravimetric analyses to account for the inorganic phase. Various magnetic measurements were conducted, including studying the field dependence of magnetisation at 10 K and 300 K within a range of 7 to −7 T. The saturation magnetisation values (Ms^300K^ and Ms^10K^) were evaluated using the following equation:

(2) $$M=M_{s}\left(1-\frac{a}{H}-\frac{b}{H^{2}}\right)$$

For H tending to ∞, the magnetisation curve was fit from 4 to 7 T [79]. The anisotropy field was calculated as a 3% difference between the magnetisation and demagnetisation curves at 10 K. The temperature dependence of magnetisation was analysed using the zero-field-cooled (ZFC) and field-cooled (FC) protocols. The sample was cooled to 5 K without any magnetic field, and then, the signals were recorded under a static magnetic field of 100 Oe. During the warm-up from 5 to 380 K, M_ZFC_ was measured, and M_FC_ was recorded during the cooling process.

## 3. Results and Discussion

**Figure 1:**
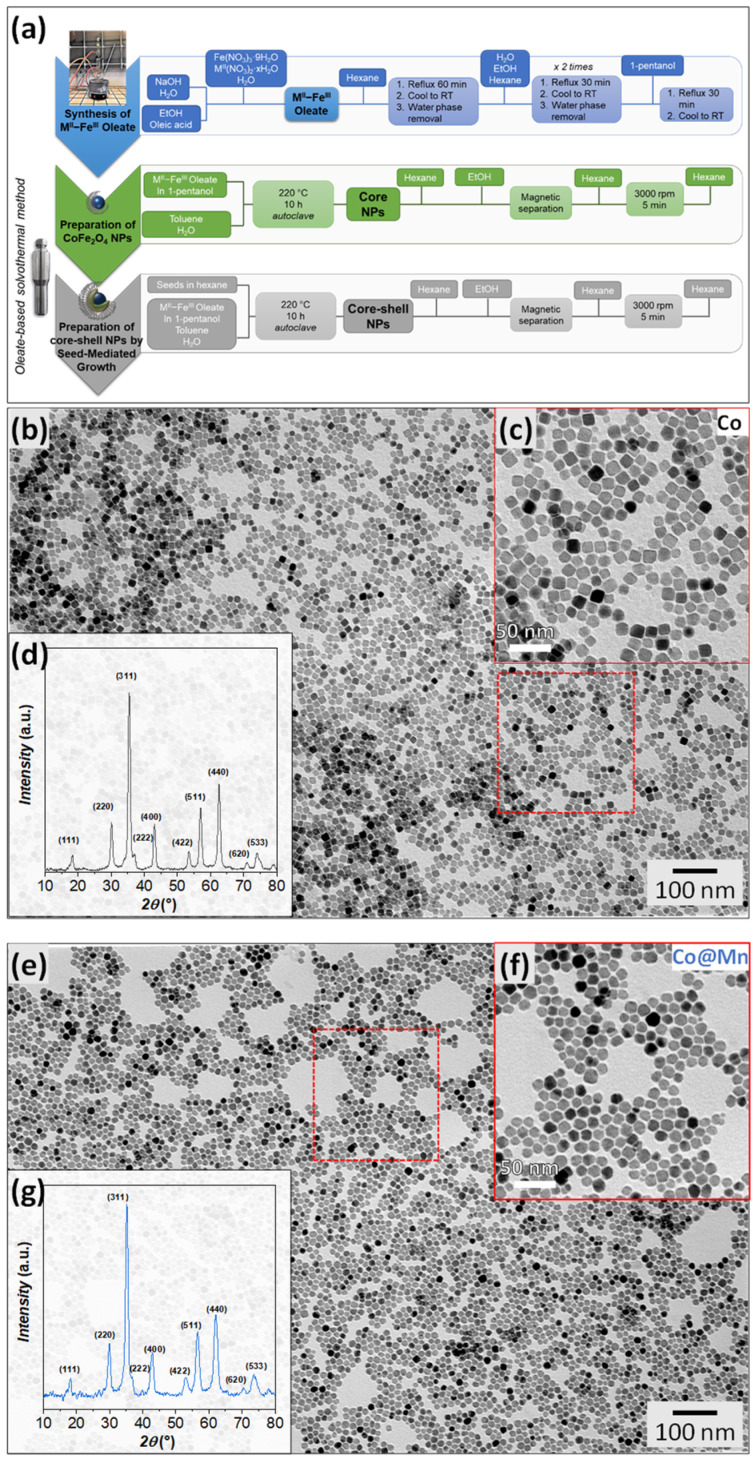
Scheme of the synthesis method: (**a**) XRD patterns and TEM images with size distributions of the Co (**b**–**d**) and Co@Mn (**e**–**g**) samples; the zones inside red boxes have been magnified as insets.

Cobalt ferrite NPs (labelled Co) with cubic shapes were obtained by following an adapted one-pot solvothermal procedure previously set up for spherical NPs [46,70,74,80,81,82], decreasing the concentration of the mixed Co-Fe oleate precursor to produce larger NPs (Figure 1, Table S1). This sample was used as seed material for the growth of a manganese ferrite shell through a second solvothermal treatment in the presence of mixed Fe^III^-Mn^II^ oleate to synthesise CoFe_2_O_4_@MnFe_2_O_4_ NPs (labelled Co@Mn). Indeed, in previous works [46,70,74], the seed-mediated growth approach in solvothermal conditions permitted the preparation of manganese-ferrite-coated cobalt ferrite NPs with different core sizes and shell thicknesses, starting from spherical particles.

The samples were characterised via FTIR and TGA to ascertain the presence of oleate molecules capping at the NPs’ surfaces (Figure S1). The FTIR spectra (Figure S1a) displayed vibrational modes of carboxylates (ν_as_(COO^−^), ν_s_(COO^−^)) at around 1550 and 1430 cm^−1^, respectively, as well as hydrocarbon chain modes in the 3000 cm^−1^ region. Thermogravimetric analyses (TGA, Figure S1b) demonstrated a comparable decrease in weight for the two samples in the temperature range of 200–350 °C, attributable to the decomposition of about 10%wt. of oleate molecules (Table 1). The slight shift in the temperature of the TGA curves and dTGA curves (Figure S1c) towards lower values for the Co@Mn sample (minima in the dTGA shifted by 15 °C) might suggest weaker bonds between the oleate molecules and the manganese cations [83], different coordinations [84], or differences in the morphological properties of the two samples (e.g., different curvature) having effects in stabilising the bonds between the carboxylate and the cations (e.g., through the proximity of the hydrocarbon chains) [85].

TEM micrographs of CoFe_2_O_4_ present well-defined cubic and spheroidal particles, quantified as about 64% and 36%, and having similar particle sizes of about 14 nm and 15 nm, respectively (Figure 1b,c and Figure S2, Table 1) with low size dispersity (σ_TEM_ = 11%, 15%). On the contrary, the TEM micrographs of the core–shell system (Figure 1e,f and Figure S3) reveal the obtainment of NPs with various shapes from spheroidal to faceted of about 15 nm (Table 1) and only a few well-defined cubic particles. EDX analysis was adopted to determine the molar ratio between the cations in the two samples. Almost stoichiometric cobalt ferrite (Co/Fe = 0.48) was obtained, in agreement with previous results on other spherical cobalt ferrite nanoparticles prepared using the same synthesis method [70]. For the core–shell sample, assuming that the cobalt content is associated with a cobalt ferrite with the molar ratio obtained for the Co sample, we can hypothesise a Mn/Fe molar ratio equal to 0.55, also revealing an almost stoichiometric manganese ferrite.

The XRD patterns of both samples show the formation of a spinel ferrite structure (Figure 1d,g). The crystallite size of Co, obtained from Rietveld refinement (Figure S4a, Table S2), is smaller (10.1(3) nm) than the mean physical size of the NPs obtained via TEM (14–15 nm, Table 1). This discrepancy might be due to the presence of structural disorder at the nanoparticle surface or to the limits of the Rietveld method in describing samples with populations of differently shaped NPs (cubic and spherical). In addition, the refinement of the Co@Mn sample using only one structural spinel phase leads to a coherent domain size smaller than the original core size (7.2(4) nm and 10.1(3) nm, respectively, Table S2). Therefore, an attempt to refine the XRD data with two spinel structures (Figure S4b, Table S3), one corresponding to CoFe_2_O_4_ and another to MnFe_2_O_4_, resulted in a better description of the XRD data. The crystallite size of the Co@Mn sample was, in this case, equal to 10.7(9) nm for the cobalt ferrite phase and 8.3(4) nm for the manganese ferrite counterpart. Moreover, the core–shell sample features a larger cell parameter (8.4030(9) Å for cobalt ferrite phase and 8.4684(7) Å for manganese ferrite) than the original core (8.3914(2) Å), in agreement with the inclusion of manganese in the spinel structure (Table 1). The adoption of the spherical harmonics function in the Rietveld refinement allowed us to visualise an average shape of the crystallites, revealing a cubic shape with rounded corners for the core sample and more faceted cuboidal particles for the core–shell one (Figure S4). The two contributions for the interpretation of the XRD pattern of the Co@Mn sample with almost constant or smaller crystallite sizes compared to the core and a slightly higher lattice parameter suggests the homogeneous nucleation of manganese ferrite, along with the formation of core–shell heterostructures with thin manganese ferrite shells, and also the occurrence of structural disorder phenomena. These findings suggest the role of the shape and the chemical nature of the metal cations involved in determining the success of the obtainment of core–shell heterostructures. To further investigate these features, HRTEM and STEM-EDX chemical mapping at the nanoscale were adopted.

**Figure 2:**
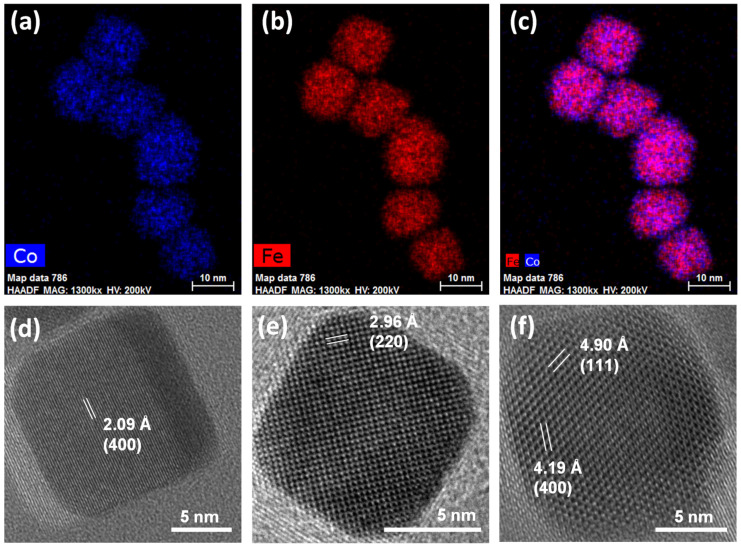
STEM-EDX chemical mapping (**a**–**c**) and HRTEM images with inter-lattice distances and Miller’s indexes of some cubic nanoparticles of the sample Co (**d**–**f**).

STEM-EDX chemical mapping and HRTEM images of the cobalt ferrite sample are reported in Figure 2a–c and Figure S5. The nanoscale chemical mapping (Figure 2a–c and Figure S5) reveals the spread of cobalt and iron throughout the particles, which appear differently oriented towards the electron beam, with hexagonal projections besides cubic ones being visible. Interestingly, the presence of the organic capping seems to be visible in the micrograph reported in Figure S5b with a size of the layer coherent with the hydrocarbon chain length [86]. HRTEM images (Figure 2d–f) show highly crystalline cubic-shaped particles with rounded corners, revealing the typical inter-lattice distances of spinel ferrite crystals and with the planes regularly aligned through the particle to the surface. The high crystallinity of the cobalt ferrite nanoparticles proved via HRTEM further suggests that the observed discrepancy between the particle and crystallite sizes is more related to the difficulties in describing two populations of differently shaped NPs via the Rietveld method.

**Figure 3:**
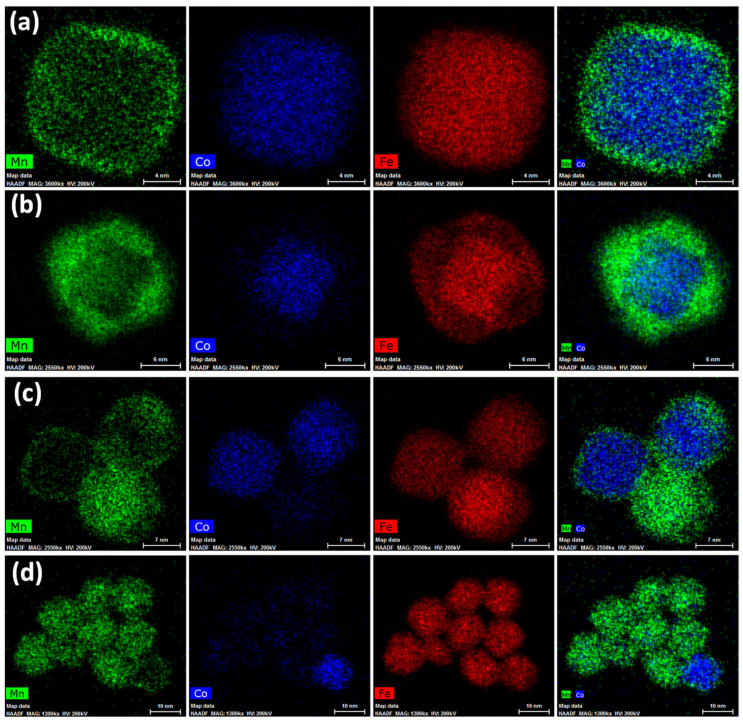
STEM-EDX chemical mapping for the sample Co@Mn, depicting core–shell heterostructures (**a**,**b**) and manganese ferrite NPs (**c**,**d**).

STEM-EDX images of the core–shell sample are shown in Figure 3, where the obtainment of core–shell NPs with very thin shells (Figure 3a,b) accompanied by the formation of manganese ferrite NPs with similar sizes and shapes (Figure 3c,d) is proved. Single-phase cobalt ferrite NPs were not detected. These results indicate that the cubic cobalt ferrite seeds of about 14 nm were homogenously coated and that the heterogeneous and homogeneous nucleations of MnFe_2_O_4_ are competitive phenomena in the selected synthetic conditions. On the contrary, the obtainment of highly crystalline NPs exclusively in a core–shell architecture was previously achieved on cobalt ferrite spherical seeds of 6 and 8 nm in size with both manganese ferrite and spinel iron oxide as the coating shell, as proven via STEM-EDX, STEM-EELS, and HRTEM [46,70]. Therefore, we can hypothesise that the bigger size as well as the faceted shape of the cobalt ferrite seeds might be the main reason for the different formation mechanism of the final products. Nevertheless, it is worth noting that the presence of uncoated seeds was never revealed in the final products obtained using this oleate-based solvothermal method, regardless of the shape and size of the seeds. As expected, at the beginning of the synthesis process, the heterogenous nucleation seems to be favoured in comparison with the homogenous nucleation. Moreover, it seems that the heterogeneous nucleation (i.e., formation of the core–shell heterostructures) goes on until a critical size is reached (about 15 nm), at which, the particles lose their colloidal stability in the liquid medium and settle at the bottom of the Teflon liner. Therefore, in the liquid medium, only Mn and Fe oleates remain, and manganese ferrite nanoparticles nucleate and grow. Figure 3a depicts cubic core–shell NPs with manganese ferrite shells of about 1.4 nm, while Figure 3b represents a core–shell nanoparticle where the manganese ferrite grew in a different direction, generating a staggered cube with respect to the cubic core. 

**Figure 4:**
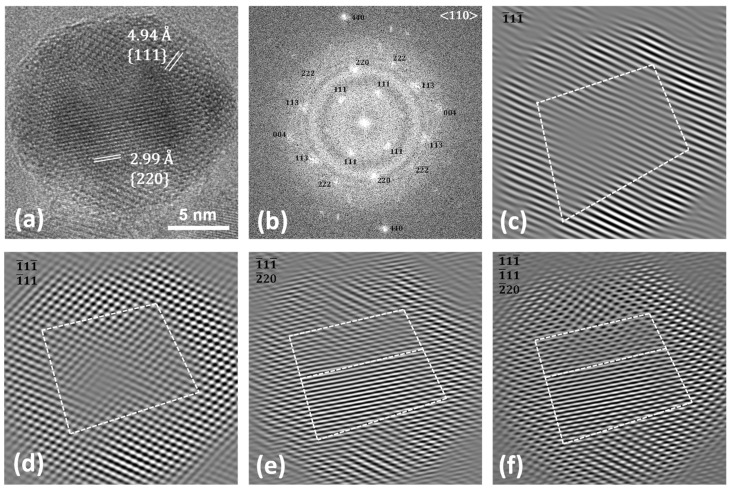
HRTEM image (**a**), FFT (**b**), and masked inverse FFT images (**c**–**f**) of Co@Mn.

**Figure 5:**
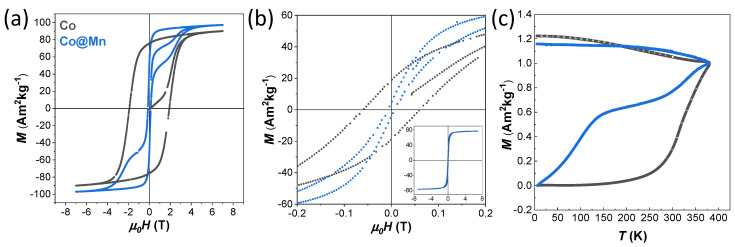
Field-dependent magnetisation curves recorded at 10 K (**a**) and 300 K (**b**), and zero-field cooled (ZFC) and field-cooled (FC) curves (**c**) of the samples Co (grey lines) and Co@Mn (blue lines).

This behaviour can also be observed in the HRTEM image of the same nanoparticle, reported in Figure 4. The particle, pointed along the <110> zone axis, reveals twins between the {220} planes of the cubic core and the {111} planes of the shell, as better evidenced in the ellipsoidal spots visible in the FFT image (Figure 4b) and in the dashed-line white squares in the inversed masked FFT images (Figure 4e,f). Figures S6 and S7 report other structural defects found for the sample. The results obtained via HRTEM and STEM-EDX agree with the scenario hypothesised for this sample on the basis of the XRD and conventional TEM data, as previously discussed. In order to verify if these compositional and structural inhomogeneities at the nanoscale level strictly related to the faceted shape of the NPs affect the macroscopic behaviour of the sample, the magnetic properties were investigated through DC magnetometry (Figure 5).

The Co sample shows hysteresis at 10 K with a large coercive field of 1.91 T and high saturation magnetisation of about 90 Am^2^kg^−1^, while the Co@Mn sample coercivity is only 0.13 T with wasp-waisted-shaped hysteresis that arises from the superposition of the hard behaviour of the core–shell architectures and the soft behaviour of the manganese ferrite NPs (Figure 5a, Table 2). This two-stage hysteresis loop at 10 K resembles those of a physical mixture, as previously observed for an ad hoc reference sample prepared by mixing two single-phase NPs (CoFe_2_O_4_ and MnFe_2_O_4_) of similar sizes (Figure 4 of [46]). A single-stage hysteresis loop with lower coercivity compared with the cobalt ferrite seeds would be instead expected as a unique contribution for core–shell heterostructures with thin shells, proving the rigid coupling between the two spinel ferrite phases. Magnetisation isotherms at 300 K (Figure 5b) still show small hysteresis with coercivity of 0.06 T for the Co sample, indicating the presence of magnetically blocked particles. Furthermore, the slightly wasp-waisted shape is still present at 300 K for the Co@Mn sample, consistent with the expected behaviour of the physical mixture of the core–shell and manganese ferrite NPs.

The temperature-dependent magnetisation curves (ZFC-FC curves of Figure 5c) confirmed the presence of magnetically blocked NPs in the two samples at room temperature (with a temperature of the maximum above 350 K), but with the presence of a shallow maximum in the ZFC curve (at about 140 K)) for the Co@Mn sample was associated with the presence of the magnetically soft MnFe_2_O_4_ NPs.

## 4. Conclusions

A one-pot solvothermal approach was exploited with the aim of obtaining cubic cobalt ferrite NPs and further coupled with a seed-mediated growth strategy to build core–shell hard–soft heterostructures. Direct proof of the obtainment of highly crystalline, cubic and spherical, stoichiometric cobalt ferrite nanoparticles was obtained via TEM, STEM-EDX, and HRTEM. These techniques, and in particular the chemical mapping at the nanoscale via STEM-EDX, allowed us to verify the production of bi-magnetic hard–soft core–shell NPs (CoFe_2_O_4_@MnFe_2_O_4_) with a very thin shell in a physical mixture with manganese ferrite NPs. Additionally, the magnetic properties were diagnostic of the superposition of two contributions: the hard behaviour of the core–shell NPs and the soft one of the manganese ferrite NPs. Indeed, a double-stage hysteresis loop in the 10 K magnetisation isotherm and a maximum in the ZFC at about 140 K associated with a magnetically soft phase were observed. These findings allowed us to hypothesise a formation mechanism in which the heterogeneous nucleation (growth of a shell on the preformed seeds) and homogeneous nucleation (formation of new particles from the metal precursors) compete. From the comparison with previous achievements in which only core–shell NPs starting from small (6–8 nm) spherical NPs were achieved, it seems that starting from bigger and faceted spinel ferrite seeds might direct the formation process of the core–shell heterostructures towards systems with very thin shells, due to a critical size being reached that made these particles no longer stable in the mother solution and made them settle down at the bottom of the synthesis reactor. Thus, the formation of new manganese ferrite nuclei occurred after this phase separation.

In this view, the obtainment of a homogenous sample of core–shell NPs might be achieved in these experimental conditions via the proper selection of the ratio between the number of preformed seeds and the metal oleate added in the seed-mediated growth process in order to avoid single-phase nucleation.

## Figures and Tables

**Table 1:** Lattice parameter (*a*), crystallite size (D_XRD_), volumetric particle size (D_TEM_V_), size distribution (σ), organic content calculated by TGA, metal atomic percentages, and molar ratio Co:Mn:Fe (with Fe moles fixed to 2) calculated via TEM-EDX of the samples. *a* and D_XRD_ values are computed via Rietveld refinement using two spinel ferrite phases for the Co@Mn sample: the first row of the table refers to CoFe_2_O_4_ and the second one to MnFe_2_O_4_.

Sample	*a* (Å)	D_XRD_ (nm)	D_TEM_V_ (nm)	σ_TEM_ (%)	Org. Phase (%wt.)	Mn (%)	Co (%)	Fe (%)	Mn:Co:Fe
Co	8.3914(2)	10.1(3)	14.2 (64% Cube)	11	10	-	32.6(7)	67.4(7)	0:0.96:2
			14.9 (36% Sphere)	15					
Co@Mn	8.4030(9) 8.4684(7)	10.7(9) 8.3(4)	15.3	12	11	25.0(2)	9.6(3)	65.4(4)	0.76:0.30:2

**Table 2:** Coercive field (H_c_) at 10 K and 300 K, anisotropy field (H_K_) at 10 K, saturation magnetisation (M_s_) at 10 K and 300 K, and remanent magnetisation (M_r_) at 10 K of the samples.

Sample	H_c_^10K^ (T)	H_K_^10K^ (T)	M_s_^10K^ (Am^2^kg^−1^)	M_r_^10K^ (Am^2^kg^−1^)	M_r_/M_s_^10K^	H_c_^300K^ (T)	M_s_^300K^ (Am^2^kg^−1^)
Co	1.91(1)	3.7(1)	92(3)	75(2)	0.82(2)	0.06(1)	77(2)
Co@Mn	0.13(1)	3.8(1)	98(3)	51(1)	0.52(2)	0.01(1)	77(2)

## Data Availability

Data are contained within the article or Supplementary Materials.

## Supplementary Materials

The following supporting information can be downloaded at: https://www.mdpi.com/article/10.3390/nano13101679/s1, Table S1: Synthesis condition for the samples; Table S2: Refined structural parameters obtained via Rietveld refinement of the XRD patterns of Co and Co@Mn samples by using one spinel ferrite phase (CoFe_2_O_4_) for both samples; Table S3: Refined structural parameters obtained via Rietveld refinement of the XRD patterns of Co and Co@Mn samples by using two spinel ferrite phases (CoFe_2_O_4_, MnFe_2_O_4_) for the Co@Mn sample; Figure S1: FTIR spectra (a), TGA curves (b), and corresponding derivatives (c) of the Co and Co@Mn samples; Figure S2: TEM micrographs of the Co sample; Figure S3: TEM micrographs of the Co@Mn sample; Figure S4: Rietveld refinement via FullProf software of the XRD patterns of Co (pattern at the bottom) and Co@Mn (pattern in the upper part) samples by using (a) one spinel ferrite phase (CoFe_2_O_4_) for both samples and (b) two spinel ferrite phases (CoFe_2_O_4_, MnFe_2_O_4_); Figure S5: STEM-EDX chemical mapping (a–c) for the sample Co; Figure S6: HRTEM images (a,c,d) of the sample Co@Mn revealing structural defects; (b) represents the masked inversed FFT image of (a); Figure S7: HRTEM micrographs (a–c) of the sample Co@Mn revealing structural defects. Reference [87] is cited in the Supplementary Materials.
