# Supplementary material for: Hard–Soft Core–Shell Architecture Formation from Cubic Cobalt Ferrite Nanoparticles
Source: Nanomaterials (Basel). 2023 May 19;13(10):1679. doi: 10.3390/nano13101679 (PMC10221987; doi:10.3390/nano13101679)
Supplement: Supplementary file 1 [file nanomaterials-13-01679-s001.zip › nanomaterials-2354735-supplementary.pdf]

Supporting Information for

Hard-soft core shell architecture formation from cubic cobalt ferrite nanoparticles

Marco Sanna Angotzi [1,2], Valentina Mameli [1,2,*], Dominika Zákutná [3], Fausto Secci [1,2], Huolin L. Xin [4] and Carla Cannas [1,2,*]

[1] Department of Chemical and Geological Sciences, University of Cagliari, Cittadella Universitaria S.P. Monserrato Sestu Km 0.700, 09042 Monserrato, Italy; marcosanna@unica.it; valentina.mameli@unica.it; fausto.secci@unica.it

[2] Consorzio Interuniversitario Nazionale per la Scienza e Tecnologia dei Materiali (INSTM), Cagliari Unit, Italy

[3] Department of Inorganic Chemistry, Charles University, Hlavova 2030, 128 40 Prague 2, Czech Republic; dominika.zakutna@natur.cuni.cz

[4] Department of Physics and Astronomy, University of California, Irvine, CA 92617; huolinx@uci.edu

* Correspondence: ccannas@unica.it; valentina.mameli@unica.it

Table S1. Synthesis condition for the samples. 2
Table S2. Refined structural parameters obtained by Rietveld refinement of the XRD patterns of Co and Co@Mn samples by using one spinel ferrite phase ($CoFe_2O_4$) for both samples. 4
Table S3. Refined structural parameters obtained by Rietveld refinement of the XRD patterns of Co and Co@Mn samples by using two spinel ferrite phases ($CoFe_2O_4$, $MnFe_2O_4$) for the Co@Mn sample. 5

Figure S1. FTIR spectra (a), TGA curves (b) and corresponding derivatives (c) of the Co and Co@Mn samples. 2
Figure S2. TEM micrographs of the Co sample. 3
Figure S3. TEM micrographs of the Co@Mn sample. 3
Figure S4. Rietveld refinement by FullProf software of the XRD patterns of Co (pattern at the bottom) and Co@Mn (pattern in the upper part) samples by using (a) one spinel ferrite phase ($CoFe_2O_4$) for both samples and (b) two spinel ferrite phases ($CoFe_2O_4$, $MnFe_2O_4$). 4
Figure S5. STEM-EDX chemical mapping for the sample Co. 6
Figure S6. HRTEM images (a, c, d) of the sample Co@Mn revealing structural defects; b) represents the masked inversed FFT image of a). 6
Figure S7. HRTEM micrographs (a, b, c) of the sample Co@Mn revealing structural defects. 7

Table S1. Synthesis condition for the samples.

Sample	n Oleate (mmol)[a]	Seeds (mg)	1-pentanol (mL)	Ethanol (mL)	Toluene (mL)	Distilled water (mL)	Temperature (°C)	Reaction time
Co	2.75	-	10	-	10	5	220	10
Co@Mn	1	20 (Co)	10	-	10	5	220	10

[a]Referred to M^{II}-Fe^{III} oleates.

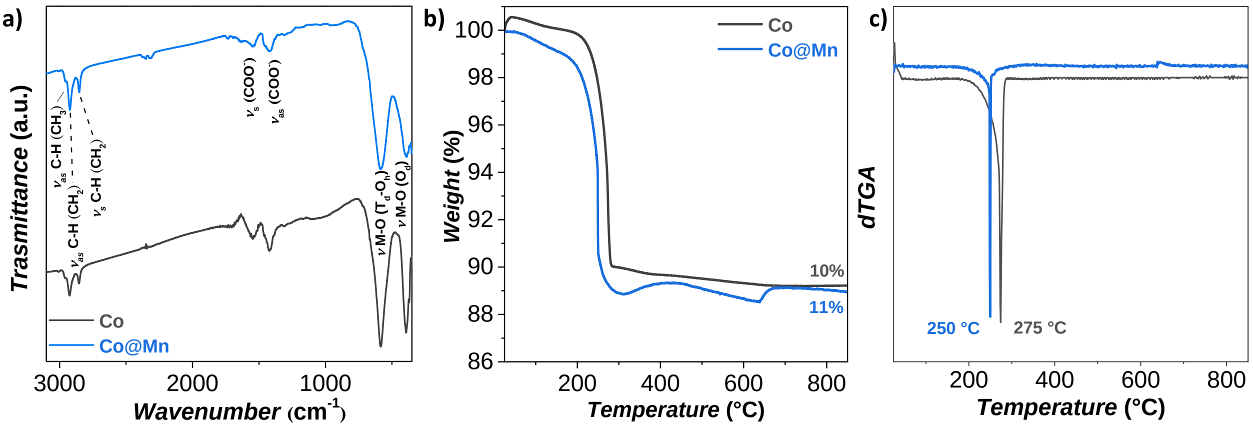

Figure S1. FTIR spectra (a), TGA curves (b) and corresponding derivatives (c) of the Co and Co@Mn samples.

FTIR spectra show the main vibrational modes related to the oleate molecule, *i.e.*, the COO^- vibrational modes (ν_{as} COO^-, ν_s COO^- at 1550 and 1420 cm^{-1}, respectively) and the ones related to the hydrocarbon chain (ν C=C, ν_{as} C-H(CH_3), ν_{as} C-H(CH_2), ν_s C-H(CH_2), ν_s C-H(CH_3), at 3007, 2956, 2924, and 2853 cm^{-1}, respectively). The bands at about 584 cm^{-1} and 394 cm^{-1} are due to the Me-O stretching mode of both the tetrahedral and octahedral sites, or the only octahedral sites of the spinel structure, respectively.[1,2]

TGA curves display a weight loss from about 200 °C till 300 °C, correspondent to the oleate decomposition.[5,6] The percentage, similar for the two samples, corresponds to a monolayer of capping molecule on the nanoparticles' surface.

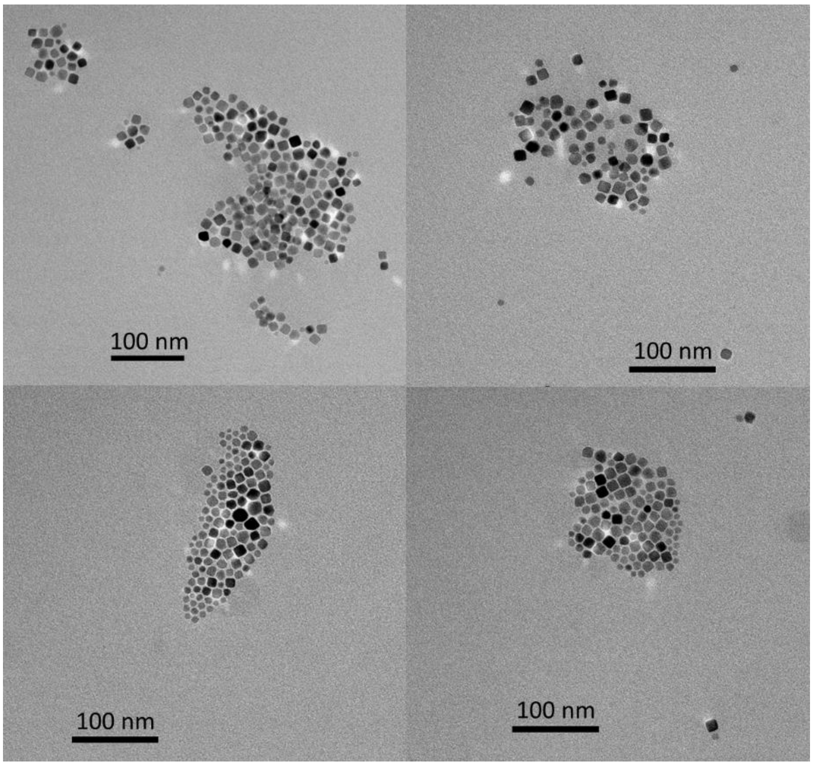

Figure S2. TEM micrographs of the Co sample.

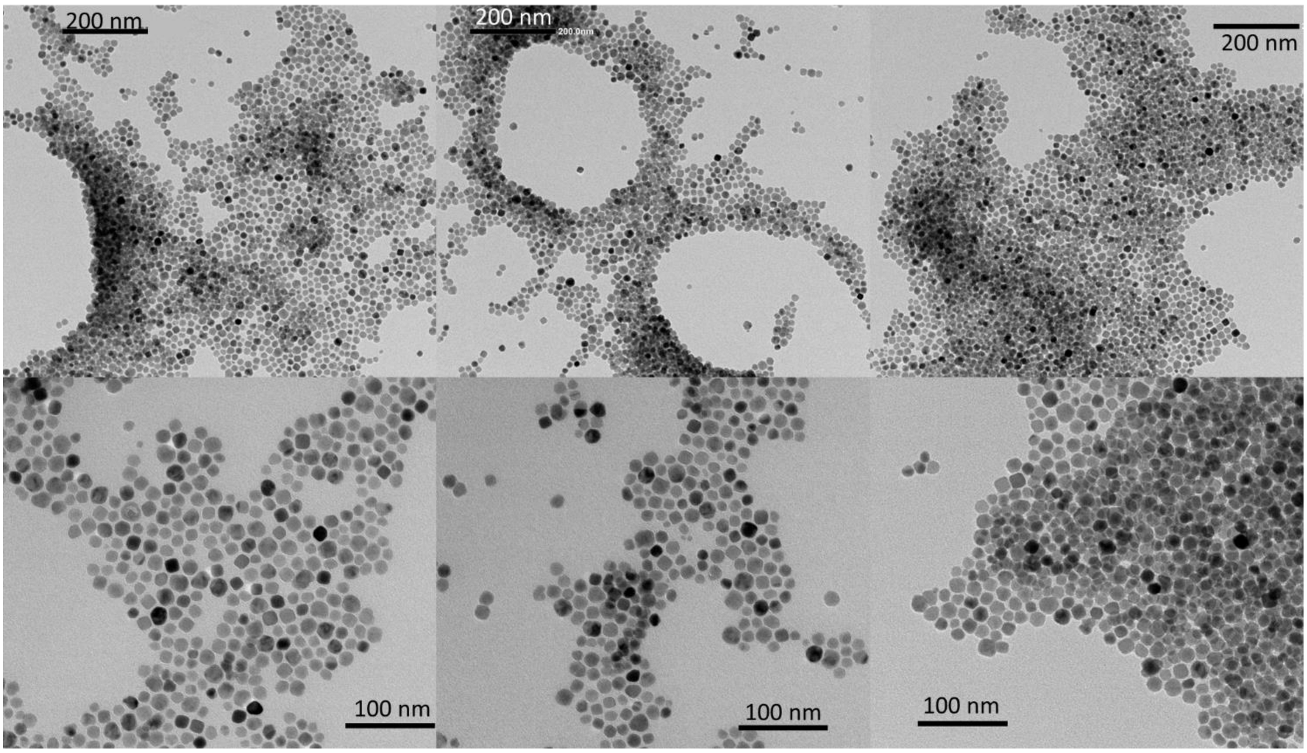

Figure S3. TEM micrographs of the Co@Mn sample.

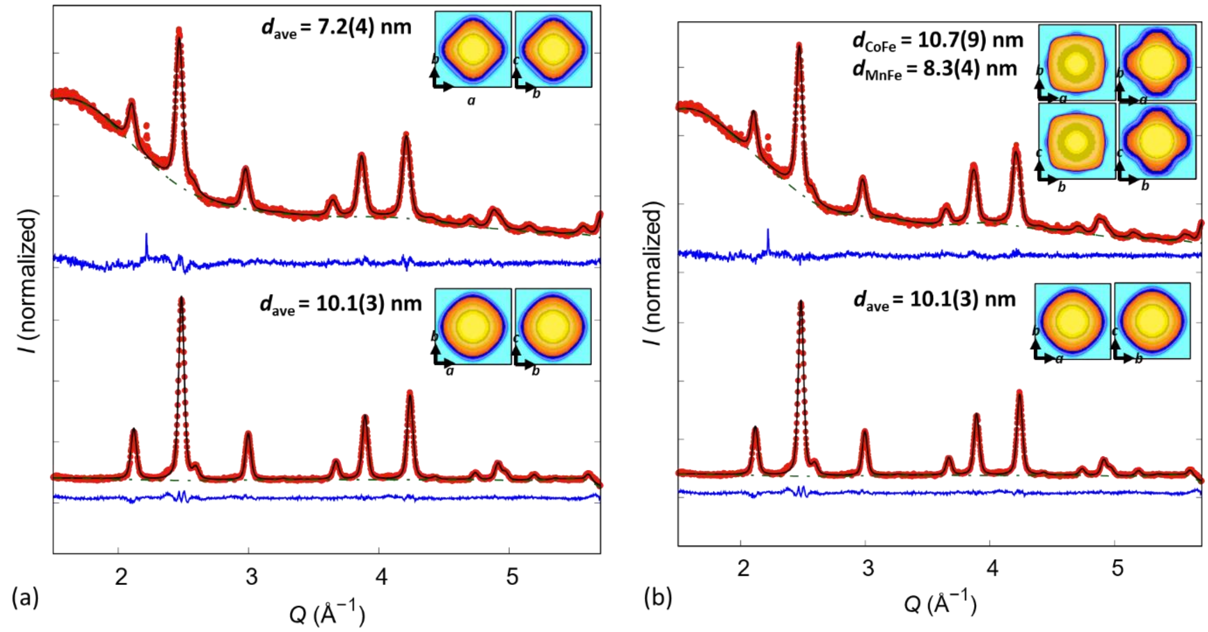

Figure S4. Rietveld refinement by FullProf software of the XRD patterns of Co (pattern at the bottom) and Co@Mn (pattern in the upper part) samples by using (a) one spinel ferrite phase ($CoFe_2O_4$) for both samples and (b) two spinel ferrite phases ($CoFe_2O_4$, $MnFe_2O_4$).

Table S2. Refined structural parameters obtained by Rietveld refinement of the XRD patterns of Co and Co@Mn samples by using one spinel ferrite phase ($CoFe_2O_4$) for both samples.

**parameter**	**MA15105**	**MA15146**
	$CoFe_2O_4$	$CoFe_2O_4$
a (A)	8.3914(2)	8.4493(5)
u (x, y, z)	0.2558(1)	0.2563(2)
profile function	Thompson–Cox–Hastings pseudo-Voigt	
BOV (Å^2)	1.30(2)	0.07(3)
G (0.01°)	0.217(2)	0.361(6)
Zero (0.01°)	0.065(1)	0.195(3)
Spherical harmonics	Laue class – m3m	
K_{00}	5.85(7)	8.8(2)
K_{41}	-0.71(8)	-1.9(1)
K_{61}	-0.04(1)	0.4(1)
K_{62}	0.00	0.00
K_{81}	-0.01(1)	0.00
Background	Chebyshev polynomial	
n	11	17
R_B (%)	2.80	3.18
R_f (%)	2.57	2.16
R_p (%)	3.61	2.76

R_{exp} (%)	3.22	1.98
χ^2	1.26	1.94

Table S3. Refined structural parameters obtained by Rietveld refinement of the XRD patterns of Co and Co@Mn samples by using two spinel ferrite phases ($CoFe_2O_4$, $MnFe_2O_4$) for the Co@Mn sample.

**parameter**	**MA15105**	**MA15146**	
	$CoFe_2O_4$	$CoFe_2O_4$	$MnFe_2O_4$
a (A)	8.3914(2)	8.4030(9)	8.4684(7)
u (*x*, *y*, *z*)	0.2558(1)	0.2561(6)	0.2566(4)
profile function	Thompson–Cox–Hastings pseudo-Voigt		
BOV (Å^2)	1.30(2)	-	-
G (0.01°)	0.217(2)	0.318(8)	0.080(5)
Zero (0.01°)	0.065(1)	0.145(3)	
Spherical harmonics	Laue class – m3m		
K_{00}	5.85(7)	3.4(4)	11.0(2)
K_{41}	-0.71(8)	-1.8(3)	1.0(2)
K_{61}	-0.04(1)	0.4(2)	-0.3(1)
K_{62}	0.00	0.00	0.00
K_{81}	-0.01(1)	0.7(1)	-1.0(1)
Background	Chebyshev polynomial		
n	11	22	
R_B (%)	2.80	2.99	2.53
R_f (%)	2.57	2.53	2.14
R_p (%)	3.61	2.25	
R_{exp} (%)	3.22	1.98	
χ^2	1.26	1.29	

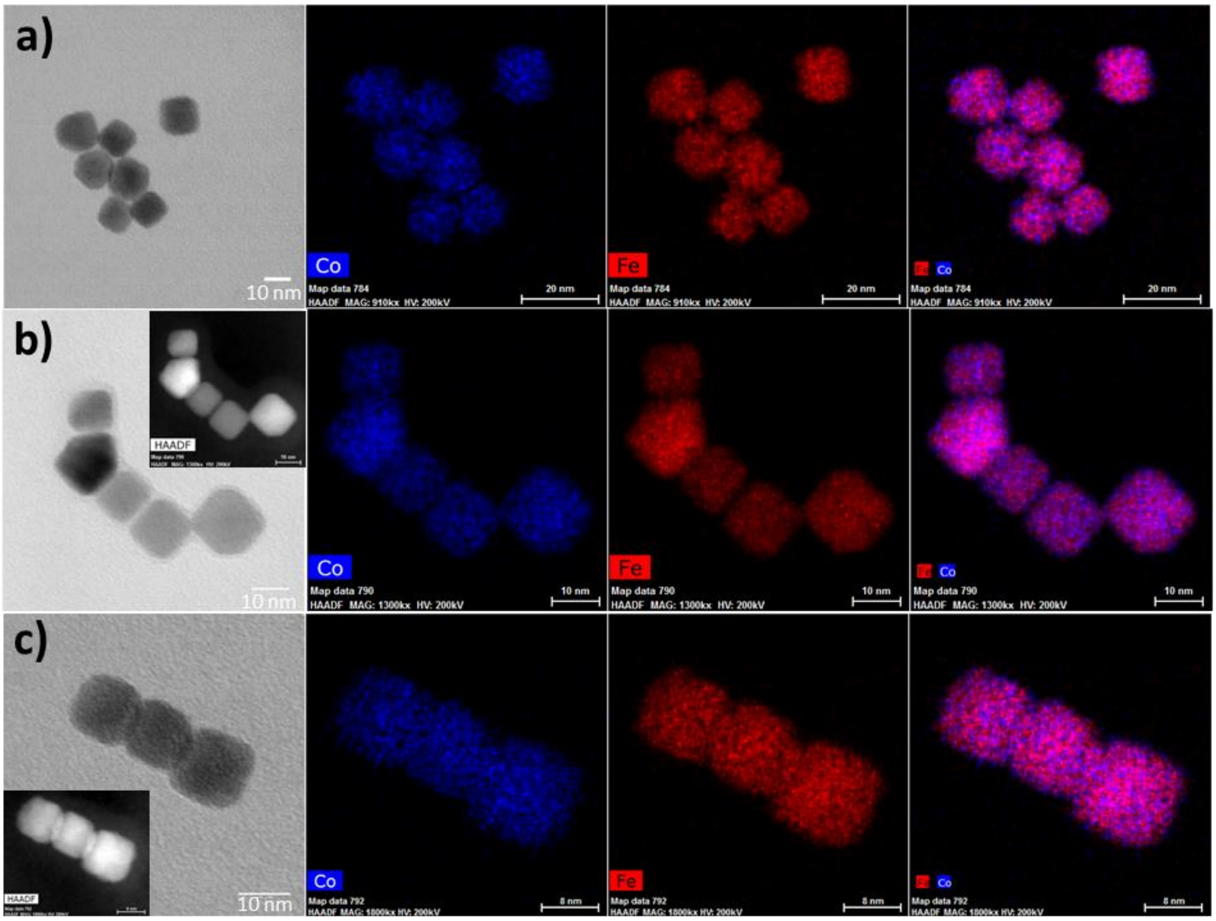

Figure S5. STEM-EDX chemical mapping (a,b,c) for the sample Co.

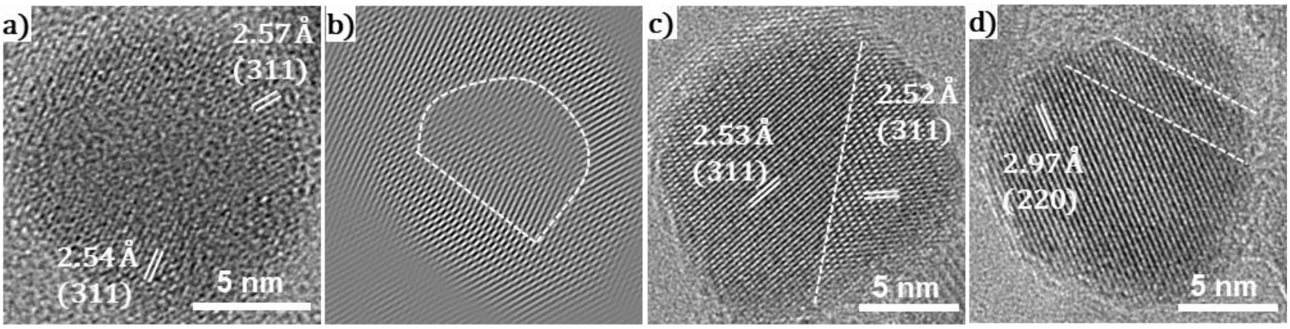

Figure S6. HRTEM images (a, c, d) of the sample Co@Mn revealing structural defects; b) represents the masked inversed FFT image of a).

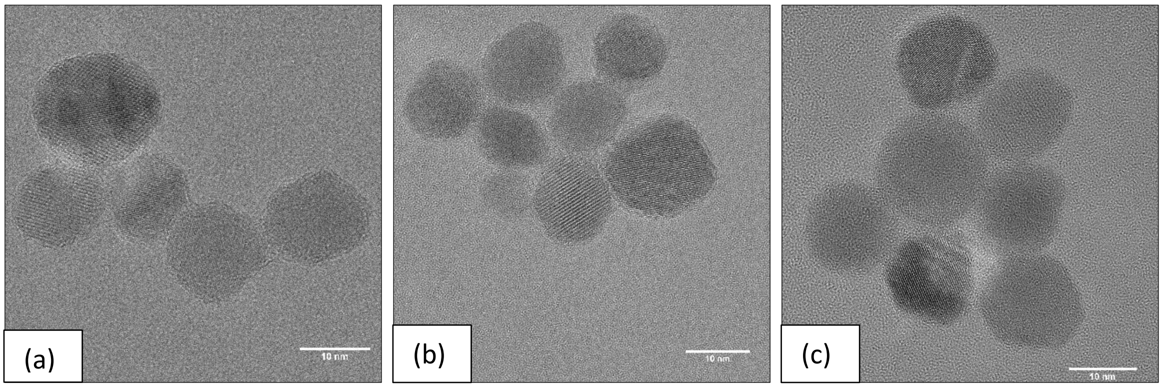

Figure S7. HRTEM micrographs (a, b, c) of the sample Co@Mn revealing structural defects.
